# Application of HPLC Combined with Laser Induced Fluorescence for Protein Profile Analysis of Tissue Homogenates in Cervical Cancer

**DOI:** 10.1100/2012/976421

**Published:** 2012-05-03

**Authors:** Sujatha Bhat, Ajeetkumar Patil, Lavanya Rai, V. B. Kartha, Santhosh Chidangil

**Affiliations:** ^1^KMC International Center, Manipal University, Manipal 576104, India; ^2^Centre for Atomic and Molecular Physics, Manipal University, Karnataka, Manipal 576104, India; ^3^Department of Obstetrics and Gynecology, Kasturba Medical College, Manipal University, Manipal 576104, India

## Abstract

A highly objective method, High Performance Liquid Chromatography with Laser Induced Fluorescence (HPLC-LIF) technique was used to study the protein profiles of normal and cervical cancer tissue homogenates. A total of 44 samples including normal cervical biopsy samples from the hysterectomy patients and the patients suffering from different stages of the cervical cancer were recorded by HPLC-LIF and analysed by Principle Component Analysis (PCA) to get statistical information on different tissue components. Discrimination of different stages of the samples was carried out by considering three parameters—scores of factor, spectral residual, and Mahalanobis Distance. Diagnostic accuracy of the method was evaluated using Receiver Operating Characteristic (ROC) analysis, and Youden's index *(J)* plots. The PCA results showed high sensitivity and specificity (*∼*100) for cervical cancer diagnosis. ROC and Youden's index curves for both normal and malignant standard sets show good diagnostic accuracy with high AUC values. The statistical analysis has shown that the differences in protein profiles can be used to diagnose biochemical changes in the tissue, and thus can be readily applied for the detection of cervical cancer, even in situations where a histopathology examination is not easy because of nonavailability of experienced pathologists.

## 1. Introduction

Cervical cancer is the second leading cause of cancer mortality in women in developing countries and seventh in developed countries [1]. In the earlier stages, the heterogeneous character of the cellular changes make correct diagnosis difficult by histopathology, since the pathologist, due to reasons like “fatigue factor” and lack of experience, may miss the “patches” in the sample [2, 3]. And the use of so-called markers, like CA 125, CEA, and so forth of no value in cervical cancer.

Optical spectroscopic methods are highly sensitive in the detection of the biochemical changes occurring in the tissue as it proceeds from normal to dysplastic and malignant conditions [4]. Many groups have studied the fluorescence spectra of cervical tissue under normal and malignant condition [5–10]. It has been shown that there are noticeable differences in the spectrum, arising from changes in tissue components [11]. In our earlier studies we have analyzed the protein profiles of serum and Pap smear in cervical malignancy, using HPLC-LIF (High Performance Liquid Chromatography-Laser Induced Fluorescence) [12–14] technique. Our system has been found to give highly reproducible protein profiles and is capable of detecting sub-femtomole quantities of proteins in 20 microlitter of a diluted sample [15]. In the present study we have used the HPLC-LIF protein profiling technique for analysis of cervical tissue samples from normal individuals and cervical cancer patients. The errors from heterogeneous nature of samples were eliminated by homogenizing the entire sample for protein profiling. Possible subjective nature of histo-pathological diagnosis is removed by rigorous mathematical/statistical pattern analysis of the protein profile to give objective diagnosis. The HPV status of the samples was not considered in the present experiments, since the main aim of the study was to see the utility of this method as a preliminary diagnostic technique in small hospitals and clinics, where facilities for such examination may not be available. Our studies show that the tissue protein profile can be used for early detection and staging of cervical malignancy with high specificity and sensitivity. The results are presented and discussed in this paper.

## 2. Methods

### 2.1. Experimental Setup

The instrumentation has been discussed in detail elsewhere [14, 15]. The HPLC system consists of an HP 1100 gradient system, Rheodyne 7725 Injection port and Biphenyl Reversed Phase narrow bore column (Vydac diphenyl, 2.1 × 250 mm, 5 *μ*m, 300 Å). Protein fluorescence was excited by irradiation of the HPLC effluent in a quartz capillary flow cell with 257.5 nm from a frequency doubled Ar^+^(Innova 90C FreD, Coherent, California, USA) laser. Protein profiles (Chromatograms) were recorded by measuring the fluorescence intensity of eluted proteins with respect to time using double monochromator (Jobin Yvon DH10 SPEX, New Jersey, USA), Chopper (EG&G model 651), Photomultiplier (Hamamatsu R 453, New Jersey, USA), and Lock-in Amplifier (EG&G model 7265) system interfaced to a computer. The experimental conditions were Laser power: 15 mW, Chopping Frequency: 20 Hz, Monochromator slits: 2 mm (Spectral band pass 8 nm), Monochromator wave length setting: 340 nm, PMT voltage: −850 volts, Lock-in Amplifier time constant: 2 seconds, and Lock in Amplifier gain: 6 dB.

### 2.2. Sample Collection and Processing

Normal tissue samples from the squamocolumnar junction of cervix were obtained from subjects who underwent hysterectomy, for reasons other than malignancy. Biopsy tissues from *cancer patients* were collected from the Department of Obstetrics and Gynecology, Kasturba Hospital, Manipal. In all cases samples were used with informed consent of subjects. The approval of the Institutional Ethics Committee was obtained for these studies (KHEC-31/2005). The *cancer patients* were at different stages of cancer of the cervix. All samples were collected from patients who came for treatment. This has resulted in availability of very few samples from stages other than II and III, for example, CIN 1, CIS, and so forth, 19 patients were in stage III, 7 in stage II, 1 stage 0 (CIN I), 1 stage IV, and 1 from dysplasia of cervix. A total of 15 normal samples and 29 malignant samples were analyzed. All the malignant samples were of squamous cell carcinoma. The sample details are given in Table 1.

All the samples, irrespective of whether they belonged to normal or *cancer patients*, were transported to the lab immediately after collection in normal saline. In the lab the tissues were washed with saline several times to remove any traces of blood. If the tissue samples were to be stored, they were immediately frozen in liquid nitrogen and stored at −80°C in the deep freeze. They were passively thawed to room temperature just before use. We have verified that this procedure did not show any noticeable difference in the protein profile of a given sample. The samples were weighed and minced with 20% wet weight of Tris-EDTA buffer. They were then homogenized by a manual homogenizer (T8 blade IKA-WERKE), centrifuged at 5000 rpm for 20 minutes twice. Supernatant was collected through a syringe fitted with 0.45 micron filter. 50 microliters of the sample homogenate was injected into the HPLC-LIF system, which had a 20 microliter loop.

### 2.3. Data Analysis

Data processing of recorded protein profiles involved background correction, smoothing, calibration, and normalization [14]. All protein profiles were normalized with respect to the 1594 seconds peak, which remained more or less constant in all samples. Data analysis was done by Principal Component Analysis (GRAMS/32, PLS PLUS/IQ software, in Galactic Corporation, USA). Diagnosis of tissue type as normal/malignant was achieved by classification of samples using Match/No Match condition of statistical parameters to those of normal and malignant calibration sets. The details of these have already been discussed in our earlier paper [14].

To start with, PCA was run with all the samples, (15 normal and 29 malignant), combined, irrespective of whether they belong to normal or malignant group. The analysis was performed using 12 factors. PCA was extended further to see whether a given tissue sample can be identified more objectively as belonging to a specific group, say, normal or malignant. This is achieved by forming calibration sets of samples certified by histopathological examination as normal or malignant, and comparing the protein profile of a test sample to each calibration set to see whether it belongs to that set or not with a given statistical probability. For this, a total of 10 samples were taken from the normal set (by random selection) to make the normal calibration set. A malignant calibration set was similarly made by taking randomly 15 samples irrespective of whether they belong to stage II or stage III samples. PCA was carried out with each of these calibration sets. The PCA scores were used to simulate the profiles of each sample and the sum of squared residuals- Σ_*p*_[*I*_*o*_^*p*^−*I*_*s*_^*p*^]^2^ calculated. Here *I*_*o*_^*p*^ and *I*_*s*_^*p*^ are the observed and simulated protein profile intensities, respectively, at point *P* on the time axis. All samples were now subjected to the Match/No match test using the three parameters, scores of factors, sum of squared residuals, and Mahalanobis distance [16]. The Mahalanobis distance is normally expressed in units of standard deviation. It is given by

(1) $$D^{2}=\left(S_{\text{test}}\right)M^{-1}{\left(S_{\text{test}}\right)}^{\prime }$$

where *S*_test_  is the vector of the scores and sum of squared residuals for a given test sample, and *M* given by *M* = ((*S*′*S*)/(*n* − 1)), where *S*  contains the corresponding parameters for the calibration set of *n* standards. 

To test whether PCA and Discriminant Analysis can be used for objective discrimination between the different stages of malignancy we have also carried out the Match/No Match test with a standard set from Stage III samples alone. 12 samples were randomly selected from the 19 stage III group and PCA was carried out with 6 factors. Though sensitivity and specificity provide a good measure of the diagnostic accuracy, it is to be noted that use of these parameters lead to conflicting demands, since to improve one, the other may have to be sacrificed. Estimating diagnostic accuracy is very important in any kind of diagnostic test, since it gives an idea of how effectively a diagnostic test can differentiate disease from normal condition. In order to arrive at the best values for sensitivity and specificity, one can apply the technique of Receiver Operating Characteristic (ROC) Curve [17]. We have carried out the estimation of the diagnostic accuracy for both normal and malignant set results by this method. One of the important measures of ROC analysis is finding Area Under the ROC-Curve (AUC), which evaluates the overall performance of the diagnostic test and is considered as the mean value of sensitivity for all the possible values of specificity [18]. The ROC curve analysis illustrates the relationship between the sensitivity and specificity of a diagnostic test. It is a measure of the performance of a diagnostic test. As already pointed out, the opposite trends of sensitivity and specificity make it difficult to arrive at suitable threshold/cutoff values for the test parameters. To remove the resulting subjectivity of choice of threshold, one can use the method of Youden's index [19]. Youden's index gives an idea about the optimum threshold/cutoff values of the test parameters used for screening. Youden's index *J*  is defined by *J* = Sensitivity + Specificity − 1. Youden's index curve is a plot of Youden's index (*J*) values vurses different operating thresholds of a test parameter (*M* distance). It shows the ideal operating point (threshold), namely, that for which *J* is maximum. At this threshold, sensitivity and specificity pairs will be having maximum values. At all other points, one or the other of these will have lower values. We have used the PCA results with normal and malignant calibration sets (i.e., Match/No Match) for these analyses. The ROC curves are plotted using specificity and sensitivity values corresponding to selected cutoff thresholds for *M* distance. The Youden's indices are calculated for different *M* distances for thresholds and plotted as Youden's indices versus thresholds.

## 3. Results

### 3.1. Visual Analysis of Protein Profiles

**Figure 1:**
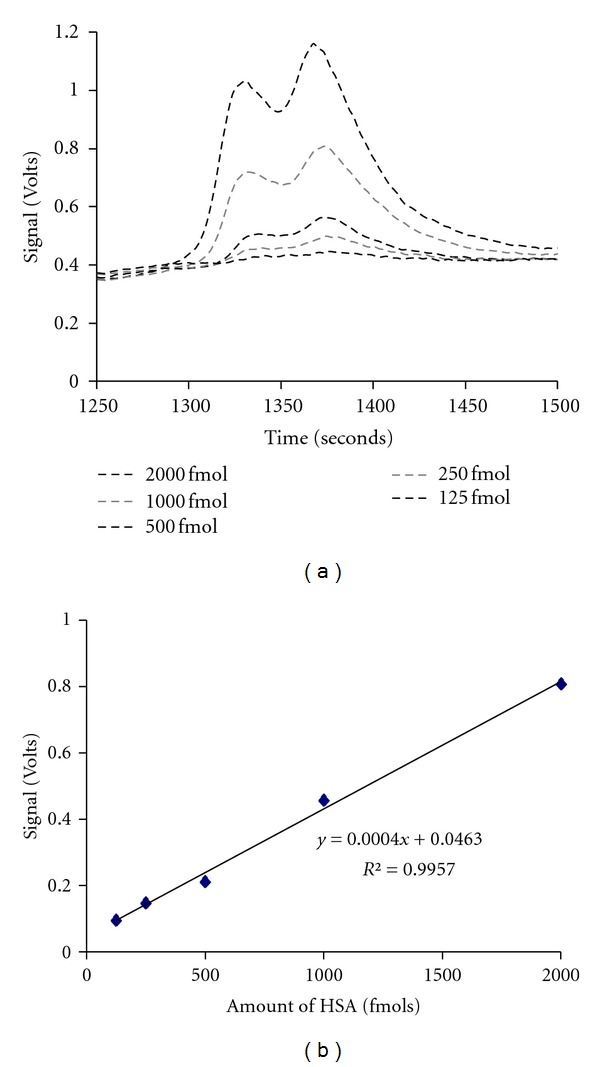
(a) Protein profiles of Human Serum Albumin (HSA) at different concentrations; (b) calibration curve for HSA.

The HPLC-LIF system used for the present study is highly sensitive, being capable of detecting trace amounts of proteins (of the order of femto moles) in microliter volume of sample. We have estimated the sensitivity of the present system by using Human Serum Albumin (HSA), a standard protein procured from Sigma Aldrich. The protein profile of Human Serum Albumin (HSA) in different concentrations and calibration graph prepared out of these data are shown in Figures 1(a) and 1(b), respectively. From the Figure 1(b), we have evaluated the limit of detection of HSA as 11.6 femtomoles.

**Figure 2:**
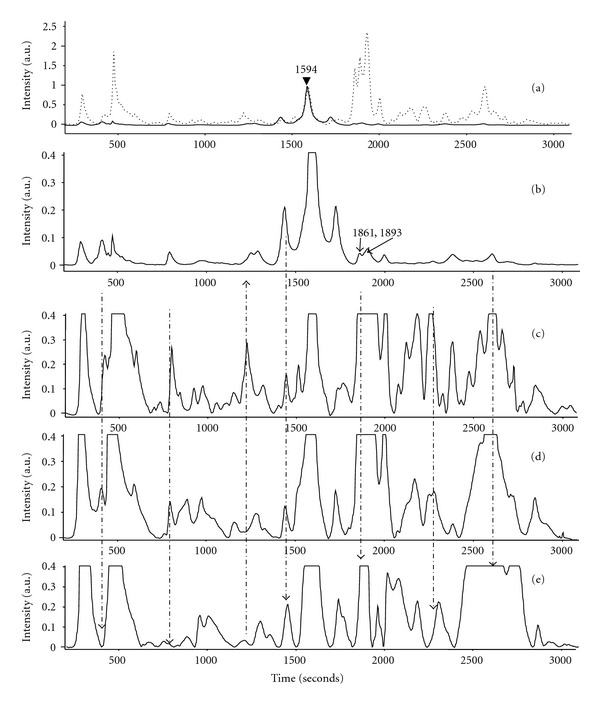
Mean protein profiles of cervical tissue homogenates: (a) normal (solid) and malignant (dotted); (b–e) expanded scale of protein profiles of tissue samples: (b) normal, (c) Stage II, (d) Stage III, and (e) Stage IV.

The mean protein profiles of the normal and malignant (stage II–IV) tissue homogenates are shown in Figure 2, illustrating the changes occurring in the protein profile as we move from normal to stage IV.

### 3.2. PCA of Combined Data

**Figure 3:**
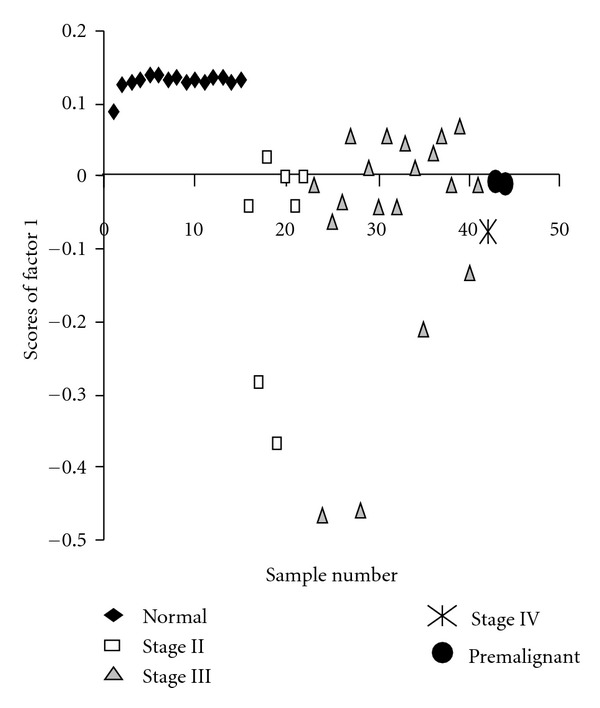
Plot of sample number versus scores of factor 1 for the combined set of samples.

Figure 3 shows the plot of sample number versus scores for factor 1 for PCA of all the samples combined. It is clear from Figure 3 that the “NORMAL” and “MALIGNANT” groups form clusters falling in different ranges of Factor 1 score. All the normal samples are having one closely spaced cluster of score values lying in between the region 0.1–1.15. Many of the malignant samples have their scores on the negative side of the plot except for 9 samples. Score values of the nine malignant samples with positive scores were found to be less, below 0.05.

As mentioned earlier, to provide a more objective diagnosis, PCA was repeated with pathologically certified calibration sets of normal and malignant samples. The results of Match/No Match with normal and malignant calibration sets are shown in Table 2. Every sample from the data set is tested for the Match/No match condition; the samples of the calibration set retrospectively (by rotating them out one by one), and all other samples prospectively by matching against the standard set. The result of PCA with a standard calibration set of Stage III samples is shown in Table 3.

### 3.3. Diagnostic Accuracy

**Figure 4:**
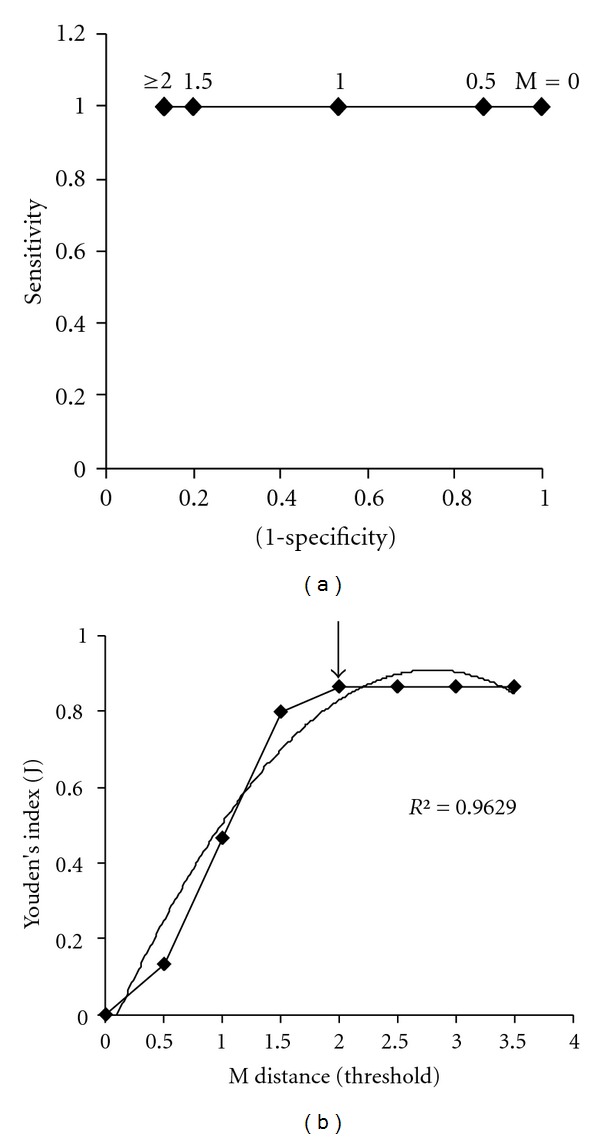
(a) Receiver Operating Characteristic (ROC) curve and (b) Youden's index curve for normal calibration set.

**Figure 5:**
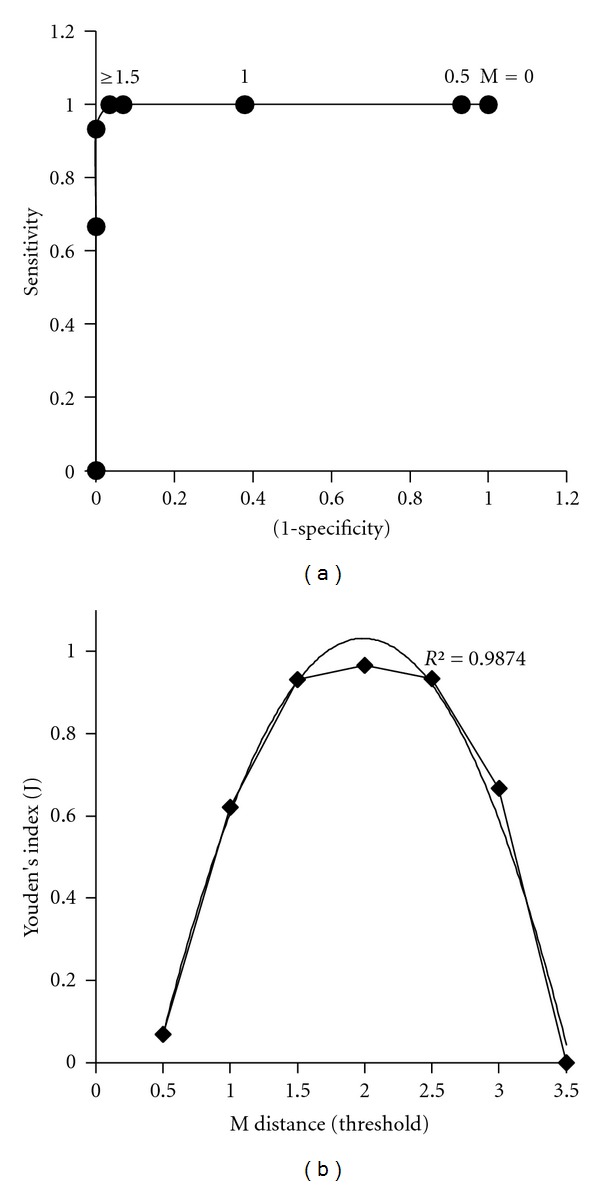
(a) Receiver Operating Characteristic (ROC) curve and (b) Youden's index curve for malignant calibration set.

Receiver Operating Characteristic (ROC) curves for normal and malignant calibration sets are shown in Figures 4(a) and 5(a), respectively. The ROC-AUC for normal and malignant sets were found to be 0.999 and 0.867, respectively. The Youden's index plots for normal and malignant calibration sets is shown in Figures 4(b) and 5(b). The optimum threshold for both calibration sets are estimated as 2  *M* distance. For higher *M* distance (*M* > 2) the results will not improve for the presented data set. Ideal operating points are marked with an arrow in Figures 4(b) and 5(b) for normal and malignant sets, respectively.

## 4. Discussion

From Figure 2 it is seen that many of the proteins present only in small amounts in the normal tissue samples are expressed much more even in the Stage II samples, and many new proteins also have appeared. As the malignancy progresses these profiles change drastically from stage II to IV giving profiles which are very different in the different stages of the disease. From the visual analysis of the protein profiles itself it is clear that many proteins which appear even in the initial 600 seconds period are expressed more (some even showing twice as intense as that of 1594 peak) compared to normal tissue. The 1861 and 1893 peaks in all the stages of the cancer are much more intensified. These and other peaks (example 250 seconds, 2600 seconds), connected with the dotted lines in Figure 2 may possibly serve as good markers, after identification, for early detection and staging. The relative intensities of these peaks are found to be almost similar to that of 1594 peak. The region from 2050–3000 seconds also shows more intense peaks.

The score values of the normal samples show that (Figure 3) at least in the age group studied; the cervical tissue has more or less very similar protein composition, irrespective of age, physiological/social condition, life style like food habits, and so forth. This provides the important possibility of identifying any change from normalcy in the cervix. The scores for the malignant group, on the other hand, are highly dispersed, presumably because of the fact that the samples are from different stages of disease. So it is clear from the plot that the score values alone can discriminate between normal and malignant samples, with a high degree of specificity and sensitivity. 

From Table 2, for the normal standard set, it is observed that except for the first two normal samples, all the other samples are showing match result. The two nonmatching normal samples though not matching with normal standard set, gave *M* distance and spectral residual much smaller than that for malignant samples. All the malignant samples, irrespective of the stage of cancer, were correctly identified as not normal, by giving FAIL result. PCA results with malignant calibration set show that, except for one, all malignant samples matched with the malignant set giving PASS result. All the normal samples, including those which were found to be not matching with the normal standard set, were found to give FAIL and did not match with the malignant standard set. The results with the normal and malignant standard set show that the method of discrimination by matching with both the calibration sets gives a very consistent diagnosis. The sensitivity of 100%, 96% and specificity of 88%, 100% were achieved by using normal and malignant standard set samples, respectively. From Table 3 it is clear that, except for five out of the 19 Stage III samples, all other samples are classified correctly using standard set of Stage III samples. All fifteen normal samples, all Stage II samples, 2 premalignant samples, and one Stage IV sample were found to give FAIL result. Though there are only few samples of early stages (CIN, CIS) in the present study, it still shows that protein profiling can discriminate these from advanced stages.

Though it is always desirable to identify and characterize the number of proteins observed in the present studies which show noticeable change from normal through various stages of malignancy, as potential tumor markers, it is very well recognized now that multiparametric protein profile analysis, may possibly be the most promising method for early detection and staging of various types of malignancies [20]. Moreover, a pattern of multiple markers can achieve a greater confidence level in early detection, staging, and followup, compared to a single marker estimation by immunoassay methods, where competing reactions as well as presence under conditions like pregnancy, hormone therapy, and so forth can mask the actual estimated amount.

## 5. Conclusions

Principal Component Analysis of protein profiles of cervical tissue samples recorded using the HPLC combined with Laser Induced Fluorescence (HPLC-LIF) technique gives very good diagnostic results. Both the standard sets from the normal and malignant samples gave consistent results. Specificity and sensitivity of the analysis are found to be very high, nearly (100%). Receiver Operating Characteristic (ROC) and Youden's index curves for both normal and malignant standard sets show good diagnostic accuracy as indicated by the high AUC values. The estimated ideal cutoff threshold is 2 *M* distance for both calibration sets. It should be mentioned here that, unlike histopathology, where heterogeneity of the tissue samples and operator subjectivity may lead to possible errors, the protein profiling of tissue samples, using optical spectroscopic methods can provide objective diagnosis of cervical cancer. Though these results have to be validated further with much larger sets of samples, the method discussed here can be adopted as a routine technique for objective diagnosis of cervical cancer.

## Figures and Tables

**Table 1:** Sample details.

S. no	Stage of cancer	Age of the patient
1–15		38–55
16–22	IIB	37–65
23–41	IIIB	35–62
42	IV	60
43	CINI	45
44	CIS	50

**Table 2:** Discriminate analysis with normal and malignant standard set.

PCA results for normal calibration set			

Sample number	Limit Tests	*M* Distance	Spec Residual

1	FAIL	79.79	22.70
2	FAIL	10.27	3.12
3–15	PASS	0.42–1.752	0.12–0.778
16–44	FAIL	95.59–3154.71	27.176–974.01

PCA results for malignant calibration set			

1	FAIL	2.017	153.097
2	FAIL	2.764	185.830
3–15	FAIL	2.97–3.32	193.60–208.19
16–33	PASS	0.42–1.24	10.04–99.47
34	FAIL	2.222	159.648
35–44	PASS	0.63–1.166	35.00–140.22

**Table 3:** Discriminate analysis with standard stage III samples.

Sample number	Limit Tests	*M* Distance	Spec Residual
1–15	FAIL	2.25–2.95	34.08−43.37
16−22 23, 24	FAIL FAIL	2.05–31.35 6.93, 24.3	34.80–422.70 104.38, 329.14
25, 26	PASS	1.05, 0.65	8.89, 7.50
27	FAIL	42.33	567.88
28−34	PASS	0.56–1.29	0.19–23.67
35	FAIL	11.49	166.55
36–39	PASS	0.65–1.59	5.95–25.8
40	FAIL	12.21	172.93
41	PASS	0.63	89.12
42–44	FAIL	2.05–13.67	36.92–194.07
